# Patient Satisfaction with Community Pharmacies Services: A Cross-Sectional Survey from Punjab; Pakistan

**DOI:** 10.3390/ijerph15122914

**Published:** 2018-12-19

**Authors:** Muhammad Majid Aziz, Wenjing Ji, Imran Masood, Muhammad Farooq, Muhammad Zubair Malik, Jie Chang, Minghuan Jiang, Naveel Atif, Yu Fang

**Affiliations:** 1 Department of Pharmacy Administration and Clinical Pharmacy, School of Pharmacy, Xi’an Jiaotong University, Xi’an 710061, China; pharmajid82@yahoo.com (M.M.A.); yfyx_8312@163.com (W.J.); jiechang@xjtu.edu.cn (J.C.); tianji712@126.com (M.J.); naveel_atif87@hotmail.com (N.A.); 2 The Center for Drug Safety and Policy Research, Xi’an Jiaotong University, Xi’an 710061, China; 3 Global Health Institute, Xi’an Jiaotong University, Xi’an 710061, China; 4 Shaanxi Center for Health Reform and Development Research, Xi’an 710061, China; 5 Department of Pharmacy, Faculty of Pharmacy and Alternative Medicine, Railway Road Campus, Islamia University, Bahawalpur 63100, Pakistan; drimranmasood@gmail.com; 6 Faculty of pharmacy, University of Lahore, Lahore 54000, Pakistan; Farooqali10@yahoo.com; 7 Department of Pharmacy, University of Sargodha, University Road Sargodha, Punjab 40100, Pakistan; Zubair_ts@yahoo.com; 8 University College of Pharmacy, University of the Punjab, Shahra-i-Quaid-e-Azam, Lahore 54000, Pakistan

**Keywords:** patient satisfaction, counseling, community pharmacy, dispensing, medicine, services, Pakistan

## Abstract

*Purpose*: Patient satisfaction can identify specific areas of improvement in community pharmacy services. Currently in Pakistan, no evidence exists in this regard. This study was conducted to determine the needs of patients and the current standards of pharmacies. *Methods*: A cross-sectional study was conducted between October 2016 and June 2017. A pilot tested questionnaire was used to collected the data from 1088 patients of 544 community pharmacies. Likert scale and one way ANOVA was used to analyze the data. *Results*: The response rate of community pharmacies was 80% and that of purchasers was 68.1%. The mean age of participants was 35.2 years. The mean overall satisfaction score of participants was 2.78/5.00. Many patients were dissatisfied (1.65/5.00) with parking facilities provided by pharmacies. Pharmacy service time fulfilled the requirements of most patients (4.16/5.00). The counseling person’s good attitude (3.99/5.00) was credited by purchasers. Level of patient satisfaction with the availability of medicines (3.19/5.00), safe storage of medicines in pharmacy stores (3.66/5.00), and quality of medicines (3.41/5.00) were almost moderate. Many patients were very satisfied (4.35/5.00) with readable instructions for their medications. Approximately half of the patients were dissatisfied with the waiting time. Many patients were also dissatisfied (2.28/5.00) with the knowledge of the counseling person. Patients perceived that staff interest in patient recovery (2.24/5.00) was low. No significant difference in level of satisfaction with regard to participant’s characteristics was found. *Conclusions*: The current study demonstrated a low level of patient satisfaction with regard to community pharmacy services in Pakistan. These services need improvement.

## 1. Introduction

Patient satisfaction is an important humanistic testimony to determine the outcome, serves and sustainability of any health care system [1]. The evidences promulgate that satisfied patients uphold good relations with their health care providers and adhere to treatment that ultimately lead to better health outcomes. More satisfied patients are persistent in using health care services and values [2].

Patient satisfaction is also a key indicator to comparing the quality of services in different patient care services, systems, and programs. These indicators are helpful for improving healthcare and for ensuring higher compliance [3]. It is also a vital tool to monitor the advancement andquality improvement in health care delivery systems [4]. Patient satisfaction studies are useful for drawing a baseline when launching new strategies [5]. An appraisal of patients’ satisfaction is necessary to optimize resource utilization [6]. Many studies have been conducted worldwide to evaluate patient satisfaction toward community pharmacy services. As this indicator become pivotal marker in developed countries, interest in patient satisfaction assessment is growing in developing countries to analyze the services of community pharmacies [2,6]. Due to difference in performance of community pharmacies, the patients’ need, perception and satisfaction level also varied in developing countries [5,6].

Similarly, the structure and operating standards of community pharmacies are at an early transition state in Pakistan. About 80% of medicines are distributed to patients through these channels [7]. There is a shortage of community pharmacists [8]. In 2011, the International Pharmaceutical Federation (FIP) and World Health Organization (WHO) jointly recommended Good Pharmacy Practice (GPP) guidelines. Together, they advised all countries to endorse GPP in community pharmacies for patients’ benefit by economical, rational, and quality use of medicines [9]. After the recommendations ofFIP and WHO, patient counseling services were started to some extent but GPP remained absolutely unnoticed; additionally, there was an urgent need for more patient-based services [10]. Doctors also have low regard for the quality of patient care in the pharmacies of Pakistan [11]. Patients are not aware about the role of pharmacists in the healthcare system [8]. Some institutional-based studies about the services of hospitals indicate that patients were not satisfied with their pharmacy services [12,13]. To the best of our knowledge, no evidence exists with regard to patient satisfaction with community pharmacy services in Pakistan. Moreover, no tool exists to analyze the patient views according current domestic situation. We designed this study to assess pharmacies services with regard to patient’s need. This work signifies the first effort in the field to evaluate patient’s satisfaction with practices and services. In addition, the current study serves as baseline data and evidence for the need to improve services and practices.

## 2. Methods

### 2.1. Study Setting

This study was conducted at selected licensed community pharmacies in Punjab, Pakistan. Punjab covers 205,344 km^2^ and is the most populous province of the country. Its population is estimated to be over 91,379,615 individuals, representing 56% of the total national population [14].

### 2.2. Selection of Pharmacy

Stratified sample technique was applied for the selection of pharmacy. A total of nine strata were formed on the basis of administrative division of government of Punjab. Each stratum was further divided in to four sub-strata: divisional city, district city, tehsil city, and suburban and rural area. 

For the selection of pharmacies, a list of pharmacies was obtained from department of health or medicine supply companies. After the licensure confirmation of each pharmacy, these were arranged geographically with a serial number. Pharmacies were systematically selected from the list by numeric selection. The sample size of pharmacies for total 22,319 pharmacies was calculated by keeping response distribution (70%), confidence interval (99%), margin of error (5%). Finally, total 544 pharmacies were selected. The participation in the study was voluntarily. In the case of denial of any pharmacy, analternative pharmacy within 2 km was selected. Thisensured a homogeneous and uniform presentation of pharmacies from all areas of Punjab province.

### 2.3. Sampling and Study Population

From our pilot study, we found that number of patients per days varies in a pharmacy. Therefore, quota sampling method was applied to find equal participation of patients from every pharmacy. Each pharmacy was focused for a single working day. Adult (aged >18 years) purchasers of medicine from community pharmacies were selected with their consent. Only the patients with a valid prescription by registered a medical practitioner, were included. The patients were approached randomly. Thus data from 1088 patients was collected that ensures frame of sampling for response distribution (50%), confidence interval (99%), margin of error (4%) for estimatedpopulation of 14,991 patients with valid prescription [14].

### 2.4. Questionnaire

The basic construct of this questionnaire was based on the key concepts of expectancy theory of Linder-Pelz [2,15] and adaptation of measuring scale was based on discrepancy theory of patients satisfaction by Fox [2,16]. To develop this tool, initially we worked to identify the domain of the constructs and item generation. For this purpose, an extensive literature survey was completed between January to April 2016. An initial pool of questions was obtained from previous studies [2,5,6,17,18,19,20,21,22,23,24,25,26,27,28,29] and available literature [30,31,32]. In addition, to determine the domains and further generation of items, a qualitative study was conducted from August to September 2016 [10]. Thus an initial version of a questionnaire of total 51 questions was completed. To check the relevance to domain, clarity and conciseness, the questionnaire was sent to experts of social and administrative pharmacy for face validity and questionnaire was modified according to suggestions. Moreover, contents were validated by a social pharmacy expert, two communitypharmacist and two general practitioners of private clinic. The ratings of each item was analyzed by an item-objective congruence (IOC) score method i.e., −1 = not representative, 0 = somewhat representative, 1 = clearly representative. The average score of IOC > 0.5 were consider good for content validity [33].

Thus a questionnaire of 44 questions was finalized.The questionnaire was originally developed in English, thentranslated into the national language (Urdu). Forward and backward translational accuracy was also ensured.The accuracy of translation was assessed by three language experts andinter-rater reliability was applied to find a proper translation. 

The further validity of instrument was assessed by a cognitive interviews method of 15 randomly selected patients [34,35,36]. A few questions found redundant or unclear to interpret, were deleted or modified. A questionnaire of 41 items was finalized.Each item was rated as relevant, understood, appropriate and not difficult and correctly interpreted by >80% of respondents of the cognitive interviews [37]. The questionnaire contained 41 questions related to patient satisfaction in four main domains: 12 questions related to the actual pharmacy store, its location, staff, and operation; fivequestions regarding medicines; 21 questions about practices; and threequestions about additional non-paid services. The patients’ demographic characteristics were also important parts of the questionnaire.

### 2.5. Pilot Study

To optimize the reliability and internal consistency of toll a pilot study of 25 patientswas conducted. The coefficient value of Cronbach’s alpha was calculated [38,39,40]. The Cronbach’s alpha of four main: domain 1 (premises and staff), domain 2 (medicines), domain 3 (dispensing and counseling practices) and domain 4 (additional non-paid services) was 0.71, 0.83, 0.75 and 0.72 respectively. Theoverall value of Cronbach’s alpha of the entire questionnaire was 0.76, giving a reasonable level of reliability and internal consistency. The data acquired for pilot study was not included in the final study results.

### 2.6. Ethics Approval and Consent to Participate

The study design and protocol was approved by the center for drug safety and policy research at the school of pharmacy after the formal approval of ethical review committee of Xi’an Jiaotong University (Ref # MR102-15/Phar)and Pharmacy Research Ethics Committee at The Islamia university Bahawalpur, Pakistan (Ref # 67-2015/PREC). The reference number (CDSP-16-PHD1-P4) was obtained before conducting study. In addition, written and verbal consent was obtained from participants and pharmacy retailers. Participants’ identities and pharmacies were anonymized. Identification numbers were used in data collection and monitoring. All participants were informed of the study purpose. 

### 2.7. Data Collection

For this cross-sectional study, data was collected by trained data collectors from October 2016 to June 2017. The participation of patients in this study was voluntarily. The questionnaire was self-administered in willing patients. However, face-to-face interviews were also conducted in the case of willing patients with low education or shortage of time. To ensure appropriate responses and quality of data, all the interviews was conducted according to the feasibility of the patient regarding location and time. Data entry was completed weekly and assessed for accuracy twice.

### 2.8. Statistical Analyses and Data Interpretation

Statistical Packages for Social Sciences version 18 (SPSS Inc., Chicago IL, US) was used to analyze the data. Specific parameters related to socio demographic characteristics and levels of satisfaction were described by calculating the average with the standard error of mean, standard deviation frequency, and percentage. Likert scale was used to determine the exact level of patients’ satisfaction. A score of 1 indicated “Not at all satisfied’, 2 denoted “Not very satisfied”, 3 indicated “Fairly satisfied”, 4 represented “Satisfied”, and 5 denoted “Very satisfied”. Then, the average mean rating of patient satisfaction was calculated.To understand the significance of variation in satisfaction level with regard to socio-demographical characteristics of the patients, one-way ANOVA was applied.

## 3. Results

The response rate of the community pharmacies was 80.0%. To meet our sample target, 682 community pharmacies were approached. Of 1597 patients, 1088 (68.1%) were ultimately available.

### 3.1. Characteristics of Participants

The mean age of the participants was 35.2 years, with most (62.1%) falling into the 26–40-year age group. The majority of participants (84.3%) were male. Many (33.8%) participants had matriculation in their formal education. 

A large proportion of participants (26.3%) earned 15,001–30,000 PkR (approximately 145–290 USD) monthly. Approximately 21.7% (237) of participants visited a pharmacy to purchase gastrointestinal medicines, as shown in Table 1, which shows the socio-demographic and financial status of participants and the disease/s for which participants visited pharmacies.

### 3.2. Level of Patient Satisfaction

The mean overall satisfaction score of participants was 2.78/5.00. According to average patients satisfaction score (3.34/5.00), the domain of medicine was ranked highest, while the domain of non-paid services was ranked lowest (2.26/5.00). The average score of domain 1 (premises and staff) and domain 3 (dispensing and counseling practices) was (2.97/5.00) and (2.87/5.00), respectively. 

#### 3.2.1. Premises and Staff 

Pharmacies were located at an average distance of 0.9 ± 0.2 km from the residences of the participants or from a hospital or clinic. The level of patient satisfaction (4.16 ± 0.82) was high for the working hours of the pharmacy. Many patients were dissatisfied with the parking facilities provided by pharmacies, with a score of 1.65/5.00, as shown in Table 2.

#### 3.2.2. Medicines

Patients appeared to be moderately satisfied (3.19 ± 0.14) with the availability of medicines or health appliances, as presented in Table 3.

#### 3.2.3. Practices

Patient satisfaction with counseling time (2.45 ± 0.38) was also low. The average counseling time of participants’ visits was 1 min and 40 s. Participants were least satisfied with dispensing times and waiting times, as shown in Table 4. During participant visits, the average dispensing time was 9.3 ± 2.3 min, while waiting time from entrance to response of staff or pharmacist (promptness) was 3.1 ± 0.52 min.

#### 3.2.4. Additional Non-Paid Services

Patients appeared to be comparatively more satisfied (3.14 ± 0.1) with on-call services, as displayed in Table 5. 

No significant difference is observed in satisfaction level of patients with respect to their demographic characteristics as shown in Table 6.

## 4. Discussion

The results of this study showed that overall satisfaction (39.6%) was low, similar to that of Portugal (39%), but it was much lower than that of Spain(76%) [22,23]. The findings of this study (2.78/5.00) range between that of Ethiopian (2.48/5.00) and Nigerian (3.02/5.00) studies [24,41]. The distinction in the level of satisfaction among different countries reflected differences in services due to a gap in the facilitating and monitoring systems [6,21,22,23,41]. The satisfaction level of participants about community pharmacy services in Punjab was low compared with that of pharmacy services in hospitals of Karachi (59.7%) and Islamabad (65%) in Pakistan [12,13]. The reason behind the low level of satisfaction can be the age of participants. The 51.6% of current study participants are below the 36 years of the age. The age of participants is one of factor of satisfaction to health services [42,43,44]. The studies [45,46] indicate that elder patients are more satisfied to health services.

The weak domains were additional services and pharmacy store, location, and staff. However, the level of satisfaction was moderate for medicine and practice standards. Most patients were dissatisfied (1.83) about the waiting area, like Ethiopian institutional pharmacies (1.53) [41]. Unlike the majority of Nigerian customers, most customers in our study were also dissatisfied with parking facilities provided by community pharmacies [25]. The operation hours of a community pharmacy was among the fundamental requirements of all participants. Participants’ views about the service time during the day were excellent and was estimated to be 11.9 ± 2.4 h, which is similar to that of independent pharmacies in Thailand (11.5 ± 2.3) [47]. A moderate perception of purchasers was found regarding the availability, quality, prices, and storage of medicines [5,24,47].

Pharmacy staff is the most important feature for execution of better services. Similar to Spanish patients, many patients or purchasers were satisfied with the attitude of pharmacy staff [26]. A moderate level of satisfaction was expressed by participants about the age of staff [21]. Many customers were discontent with the qualification of pharmacy staff and the knowledge of the counseling person [2,24,42]. Therefore, participants were not pleased with counseling services. Participants’ views about counseling services at community pharmacies in Pakistan (45.6%), the Netherlands (42.0%), and South Korea (42.0%) are indistinct [27,28]. Optimal counseling can improve patient compliance to medication [2,31], but like Saudi Arabians, approximately half of participants perceived that the necessary instructions and warnings for medications were not conveyed [5]. Thus, knowledge of the counseling person and counseling services should be improved to fulfill patients’ needs. This study declares that medication information should be clearer and standardized. In addition, the average counseling time reported by purchasers was 1.4 min compared with the time reported by South Korean patients (<1 min). Increasing counseling duration would lead to imparting essential information to patients, which would thereby significantly increase patient contentment [27].

Patients cannot afford to wait a long time to receive necessary services due to poor health or life-threatening emergencies. Many purchasers, like Estonians, felt discomfort due to long waiting times [29]. Therefore, fulfillment of prescriptions was reported to be slow by participants [24,25]. Patients reported that dispensing time was also excessive. However, patients have moderate views about dispensing patterns, and the findings are indistinguishable from previous results from Thailand and Estonia [29,42].

Community pharmacies are ideally located to promote health awareness by providing information about ongoing health camps and campaigns [32]. However, identical to findings in Saudi Arabia, this study divulged that the least informative services were provided by pharmacies [5]. On the basis of patient’s views, we perceived that additional or non-paid facilities that were offered to them were better than that of Nigerian pharmacies but lower than those in Thailand [25,42]. Thus, the overall performance of community pharmacies was good. Community pharmacy services need many improvements and modifications in their basic structure and function. The Ministry of Health, officials, and authorities can strengthen pharmacy care services by implementing appropriate policies and encouraging pharmacy staff training.

## 5. Limitations

Firstly, this report shows only the views of agreed and selected patients from selected pharmacies. Minor dissimilarities in the selection of different patients from different pharmacies in different localities may possible. To overcome, such a type of exaggeration of respondent, we applied a 5 pointLikert scale. Secondly, this study did not focus on the the reasonsforpatients’ refusal to participate in the study. Thirdly, this study did not provide any insight into participants’ severity of disease. Fourthly, patients having valid prescriptions were included in this study. The views of patients purchasing OTC medicines may differ. Fifthly, most study participants were male due to the socio-cultural norms of the study setting.

## 6. Conclusions

The current study demonstrated low patient satisfaction with regard to community pharmacy services in the Punjab region of Pakistan. This result showed that community pharmacy services are comparatively poorer than the pharmacy services offered at hospitals due to inappropriate policies and weak execution. The low level of satisfaction may attribute to the young participation. Community pharmacy services need to be improved. Continuous professional training programs for pharmacy staff are also needed. In addition, authorities should focus on and play a role in strengthening pharmacy care services.

## Figures and Tables

**Table 1:** Characteristics of study participants (*n* = 1088).

Characteristics	Range/Groups	Frequency	Percentage
Age (years)	25 or below	93	8.5
	26–30	181	16.6
	31–35	289	26.5
	36–40	207	19.0
	41–45	168	15.4
	46–50	103	9.4
	51 or above	47	4.3
Mean age ± SD		35.2 ± 3.6	
Educationalstatus	Below matriculation	44	4.0
	Matriculation	368	33.8
	Intermediate	219	20.1
	Bachelor	259	23.8
	Master	183	16.8
	Higher Education	15	1.4
Gender	Male	917	84.3
	Female	171	15.7
Monthly income inPakistani rupees (PkR)	Less then or 15,000	251	23.1
	15,001–30,000	286	26.3
	30,001–45,000	216	19.8
	45,001–60,000	249	22.8
	More then 60,000	86	7.9
Disease (for which participant visited pharmacy)	Gastrointestinal diseases	237	21.7
	Respiratory infections	212	19.4
	Cardiac diseases	198	18.2
	Orthopedic problems	146	13.4
	Urinary tract infections	123	11.3
	Eye diseases	94	8.6
	Dermatological problems	71	6.5
	Oncological diseases	5	0.4
	Other diseases	2	0.2

**Table 2:** Patients’ satisfaction about the shop, location and staff (*n* = 1088).

Measures	Not at All Satisfied *n* (%)	Not Very Satisfied *n* (%)	Fairly Satisfied *n*(%)	Satisfied *n* (%)	Very Satisfied *n* (%)	MS ± SEM
Location of pharmacy is suitable for you?	136 (12.5)	371 (34.1)	306 (28.1)	178 (16.3)	96 (8.8)	2.74 * ± 0.03
Are you satisfied about dispensing area in pharmacy shop?	91 (8.3)	187 (17.2)	146 (13.4)	449 (41.2)	215 (19.7)	3.46 ± 0.03
Do you feel the comfort and convenience at the waiting area of pharmacy?	509 (46.8)	364 (33.4)	117 (10.7)	86 (7.9)	12 (1.1)	1.83 ± 0.02
Are you satisfied about counseling area of pharmacy shop? (noise free/ separate)	509 (46.7)	348 (31.9)	129 (11.8)	91 (8.3)	11 (1.0)	1.84 ± 0.03
Are you satisfied with cleanliness and hygienic condition of pharmacy shop?	106 (9.7)	258 (23.7)	182 (16.7)	418 (38.4)	124 (11.4)	3.18 ± 0.03
Are you satisfied to parking facility provided by pharmacy?	701 (64.4)	201 (18.4)	87 (7.9)	62 (5.7)	37 (3.4)	1.65 ± 0.03
Are you satisfied by operational hours of pharmacy?	12 (1.1)	51 (4.68)	36 (3.3)	634 (58.3)	355 (32.6)	4.16 ± 0.02
Are you satisfied to pharmacy operational hours at weekends and public holidays or festivals?	28 (2.5)	183 (16.8)	276 (25.3)	456 (41.9)	145 (13.3)	3.46 ± 0.03
Do you think staff is well educated?	315 (28.9)	291 (26.7)	287 (26.3)	180 (16.5)	15 (1.3)	2.34 ± 0.03
Are you satisfied with the staff attitude?	47 (4.3)	21 (1.9)	128 (11.7)	678 (62.3)	214 (19.6)	3.91 ± 0.02
Are satisfied with age of pharmacy staff?	34 (3.1)	17 (1.5)	145 (13.3)	714 (65.6)	178 (16.3)	3.90 ± 0.02
Are you satisfied that number of staff is adequate to pharmacy operational requirement?	89 (8.1)	196 (18.0)	191 (17.5)	578 (53.1)	34 (3.1)	3.25 ± 0.03

* 1 participant didn’t respond this question.

**Table 3:** Patients’ satisfaction about the medicine handling (*n* = 1088).

Measures	Not at All Satisfied *n* (%)	Not Very Satisfied *n* (%)	Fairly Satisfied *n* (%)	Satisfied *n* (%)	Very Satisfied *n* (%)	MS ± SEM
Are you satisfied with availability of medicines or health appliances you need?	154 (14.1)	89 (8.2)	332 (30.5)	412 (37.8)	101 (9.2)	3.19 ± 0.03
Are satisfied that you receive the medications from the pharmacy exactly according to the prescription or need?	146 (13.4)	221 (20.3)	250 (22.9)	389 (35.7)	82 (7.5)	3.03 ± 0.03
Are you satisfied with appropriate and safe storage of medicines in pharmacy shop or its ware-house?	78 (7.1)	154 (14.1)	132 (12.1)	412 (37.8)	312 (28.6)	3.66 ± 0.03
Are you satisfied with the quality of medicines?	37 (3.4)	137 (12.5)	339 (31.1)	486 (44.6)	89 (8.2)	3.41 ± 0.02
Are you satisfied with prices or discounts on medicines or health appliances you need?	55 (5.1)	161 (14.7)	259 (23.8)	506 (46.5)	107 (9.8)	3.41 ± 0.03

**Table 4:** Patients’ satisfaction withthe dispensing and counseling practices (*n* = 1088).

Measures	Not at All Satisfied *n* (%)	Not Very Satisfied *n* (%)	Fairly Satisfied *n* (%)	Satisfied *n* (%)	Very Satisfied *n* (%)	MS ± SEM
Are you satisfied with dispensing time?	415 (38.1)	199 (18.2)	305 (28.0)	133 (12.2)	36 (3.3)	2.24 ± 0.03
Are you satisfied that the pharmacist/staff consult and cooperate with the physician for the correct dispensing of medicines to you?	268 (24.6)	205 (18.8)	337 (30.9)	256 (23.5)	22 (2.0)	2.59 ± 0.03
Are you satisfied with dispensing pattern like care and proper labeling?	191 (17.5)	71 (6.5)	429 (39.4)	165 (15.1)	232 (21.3)	3.16 ± 0.04
Are you satisfied that instructions on your medications are easily readable?	13 (1.2)	8 (0.7)	11 (1.0)	607 (55.7)	449 (41.2)	4.35 ± 0.02
Do you afford waiting time?	415 (38.1)	198 (18.2)	313 (28.7)	129 (11.8)	33 (3.0)	2.23 ± 0.03
Are you satisfied with attitude of counseling person?	14 (1.3)	15 (1.3)	231 (21.2)	526 (48.3)	302 (27.7)	3.99 ± 0.02
Are you satisfied with knowledge of counseling person?	401 (36.8)	213 (19.5)	289 (26.5)	134 (12.3)	51 (4.6)	2.28 ± 0.03
Are you satisfied with the communication method of counseling person was effective enough?	26 (2.4)	22 (2.0)	357 (32.8)	564 (51.8)	119 (10.9)	3.66 ± 0.02
Are you satisfied with counseling time?	373 (34.2)	182 (16.7)	261 (23.9)	214 (19.6)	58 (5.3)	2.45 ± 0.03
Are you satisfied with counseling without request?	9 (0.8)	12 (1.1)	589 (54.1)	167 (15.3)	311 (28.5)	3.69 ± 0.02
Are you satisfied with additional counseling on request or re- counseling (if requested, they will provide)?	13 (1.2)	35 (3.2)	345 (31.7)	578 (53.1)	117 (10.7)	3.69 ± 0.02
Are you satisfied with privacy for discussions?	287 (26.3)	224 (20.5)	308 (28.3)	218 (20.0)	51 (4.6)	2.56 ± 0.03
Are you satisfied about storage information of your medication, provided by pharmacy (if provided any)	204 (18.7)	278 (25.5)	355 (32.6)	244 (22.4)	7 (0.6)	2.6 ± 0.03
Are you satisfied about enquiries of compliance to the previously dispensed prescription? (if provided any)	372 (34.2)	136 (12.5)	295 (27.1)	234 (21.5)	51 (4.6)	2.5 ± 0.03
Did they provide you any knowledge to dietary compliance regarding your disease? (if provided any)	256 (23.5)	211 (19.3)	489 (44.9)	89 (8.1)	43 (3.9)	2.49 ± 0.03
Are you satisfied about the provided knowledge for physical exercise regarding your health? (if provided any)	117 (10.7)	258 (23.7)	186 (17.1)	378 (34.7)	149 (13.6)	3.16 ± 0.03
Are you satisfied about the knowledge provided for smoke cessation regarding your health? (if provided any)	256 (23.5)	277 (25.4)	248 (22.7)	173 (15.9)	134 (12.3)	2.68 ± 0.04
Are you satisfied by the interest of pharmacy staff in your medical conditions improvement or any complication and disease controlled?	364 (33.4)	307 (28.2)	217 (19.9)	187 (17.1)	13 (1.2)	2.24 ± 0.03
Are you satisfied with necessary instructions and warnings about your medications (side effects, drug-drug interactions, food and drug interactions), especially for medications received for the 1st time?	341 (31.3)	211 (19.4)	289 (26.5)	188 (17.2)	59 (5.4)	2.46 ± 0.03
Are you satisfied thatpharmacist or staff explains the treatment periodsufficiently (especially when you receive a medication for the 1st time)	354 (32.5)	189 (17.3)	311 (28.5)	119 (10.9)	115 (10.5)	2.49 ± 0.04
Are you satisfied that pharmacist or staff tries to make sure that you understand how to take your medications properly?	156 (14.3)	198 (18.1)	415 (38.1)	213 (19.5)	106 (9.7)	2.92 ± 0.03

**Table 5:** Patients; satisfaction withadditional non-paid services (*n* = 1088).

Measures	Not at All Satisfied *n* (%)	Not Very Satisfied *n*(%)	Fairly Satisfied *n* (%)	Satisfied *n* (%)	Very Satisfied *n* (%)	MS ± SEM
Are you satisfied about services in emergency out of operational time of pharmacy (on call services)?	19 (1.7)	314 (28.8)	367 (33.7)	264 (24.2)	124 (11.3)	3.14 ± 0.03
Are you satisfied about informative services about the ongoing health camps or campaigns e.g., Polio eradication, free medication e.g., Free TB medication in your locality, possible drug shortage or price increase and decrease in future	418 (38.4)	277 (25.4)	144 (13.2)	187 (17.1)	62 (5.6)	2.26 ± 0.03
Are you satisfied with non-paid facilities like blood pressure, weighing machine, home delivery or any other?	211 (19.3)	376 (34.5)	269 (24.7)	166 (15.2)	66 (6.0)	2.54 ± 0.03

**Table 6:** Difference in the mean level of satisfaction by characteristics of study participants (result of one-way ANOVA).

Characteristics	Range/Groups	Mean (SD)	*p*-Value
Age (years)	25 or below	2.50 (0.530)	0.382
	26–30	3.22 (0.705)	
	31–35	3.13 (0.585)	
	36–40	3.11 (1.237)	
	41–45	3.03 (0.120)	
	46–50	2.94 (0.680)	
	51 or above	2.59 (0.825)	
Educationalstatus	Below matriculation	2.97 (0.544)	0.276
	Matriculation	2.69 (0.642)	
	Intermediate	3.15 (0.766)	
	Bachelor	3.00 (0.714)	
	Master	2.97 (0.077)	
	Higher Education	4.35 (0.680)	
Gender	Male	2.93 (0.678)	0.932
	Female	2.99 (0.733)	
Monthly income inPakistani rupees (PkR)	Less then or 15,000	2.74 (0.536)	0.953
	15,001–30,000	3.13 (0.777)	
	30,001–45,000	3.28 (0.771)	
	45,001–60,000	2.63 (0.585)	
	More then 60,000	3.02 (0.710)	
Disease (for which participant visited pharmacy)	Gastrointestinal diseases	2.81 (0.674)	0.864
	Respiratory infections	2.97 (0.610)	
	Cardiac diseases	3.03 (0.781)	
	Orthopedic problems	3.11 (0.971)	
	Urinary tract infections	3.13 (0.725)	
	Eye diseases	2.34 (.473)	
	Dermatological problems	2.82 (0.456)	
	Oncological diseases	2.59 (0.683)	
	Other diseases	2.23 (0.758)	
